# (Ga_0.71_B_0.29_)PO_4_ with a high-cristobalite-type structure refined from powder data

**DOI:** 10.1107/S1600536810000358

**Published:** 2010-01-09

**Authors:** Ya-Xi Huang, Jin-You Liu, Jin-Xiao Mi, Jing-Tai Zhao

**Affiliations:** a Department of Materials Science and Engineering, College of Materials, Xiamen University, Xiamen 361005, People’s Republic of China; b Chinese Academy of Sciences, Shanghai Institute of Ceramics, State Key Laboratory of High Performance Ceramics and Superfine Microstructure, 1295 Dingxi Road, Shanghai 200050, People’s Republic of China

## Abstract

Gallium boron phosphate, (Ga_0.71_B_0.29_)PO_4_, was synthesized by a high-temperature solid-state reaction method. The crystal structure is isostructural with the tetra­gonal high-cristobalite structure with space group *P* 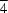 which is built from alternating Ga(B)O_4_ and PO_4_ tetra­hedra inter­connected by sharing the common O-atom vertices, resulting in a three-dimensional structure with two-dimensional six-membered-ring tunnels running along the *a* and *b* axes.

## Related literature

For information on cristobalite structures, see: Achary *et al.* (2003 ▶). For borophosphate structures, see: Ewald *et al.* (2007 ▶); Mi *et al.* (1999 ▶); Schmidt *et al.* (2004 ▶); Schulze (1934 ▶); Dachille & Glasser (1959 ▶); Mackenzie *et al.* (1959 ▶). For the catalytic properties of BPO_4_, see: Moffat (1978 ▶); Moffat & Schmidtmeyer (1986 ▶); Mooney (1956 ▶); Morey *et al.* (1983 ▶); Tada *et al.* (1987 ▶); Tartarelli *et al.* (1970 ▶). For crystallographic background, see: Finger *et al.* (1994 ▶)); Thompson *et al.* (1987 ▶).

## Experimental

### 

#### Crystal data

(Ga_0.71_B_0.29_)PO_4_
*M* *_r_* = 147.61
Tetragonal, 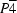

*a* = 4.7343 (1) Å
*c* = 7.0896 (4) Å
*V* = 158.90 (1) Å^3^
*Z* = 2
Cu *K*α_1_, Cu *K*α_2_ radiation
λ = 1.5405, 1.5443 Å
*T* = 293 K
flat sheet, 10 × 10 mm

#### Data collection

Rigaku-D/max automatic powder diffractometer
Specimen mounting: packed powder pellet
Data collection mode: reflection
Scan method: step
2θ_min_ = 15.03°, 2θ_max_ = 100.02°, 2θ_step_ = 0.01°

#### Refinement

*R*_p_ = 0.076
*R*_wp_ = 0.129
*R*_exp_ = 0.072
*R*(*F* ^2^) = 0.07586
χ^2^ = 3.204
8500 data points
35 parameters
2 restraints

### 

Cell refinement: *GSAS* (Larson & Von Dreele, 2004 ▶); data reduction: *GSAS*; program(s) used to refine structure: *GSAS* (Larson & Von Dreele, 2004 ▶); molecular graphics: *DIAMOND* (Brandenburg, 2005 ▶); software used to prepare material for publication: *GSAS*.

## Supplementary Material

Crystal structure: contains datablocks global, I. DOI: 10.1107/S1600536810000358/br2131sup1.cif

Rietveld powder data: contains datablocks I. DOI: 10.1107/S1600536810000358/br2131Isup2.rtv

Additional supplementary materials:  crystallographic information; 3D view; checkCIF report

## Figures and Tables

**Table 1:** Selected bond lengths (Å)

(Ga/B)1—O2^i^	1.7079 (5)
(Ga/B)2—O1^ii^	1.6979 (5)
P1—O1	1.499 (3)

Symmetry codes: (i) 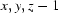; (ii) 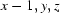.

**Fig. 1.**
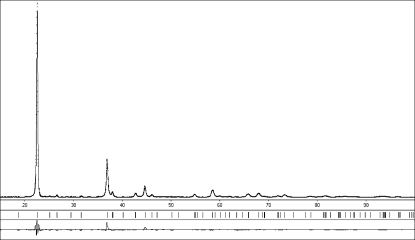
Experimental (points) and calculated (lines) X-ray diffraction patterns of (Ga0.71B0.29)PO4. The difference profile is given at the bottom. The Bragg positions are indicated by the vertical marker below the observed pattern.

**Fig. 2.**
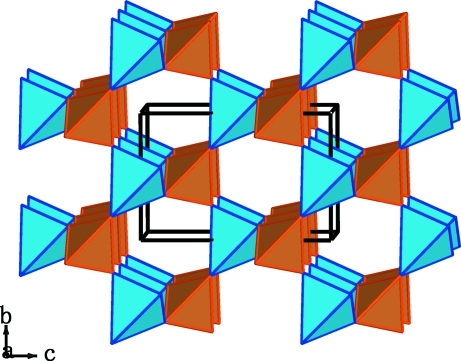
The crystal structure of (Ga0.71B0.29)PO4 viewed along the a-axis. (Ga,B)O4 tetrahedra: blue, PO4 tetrahedra: orange.

**Fig. 3.**
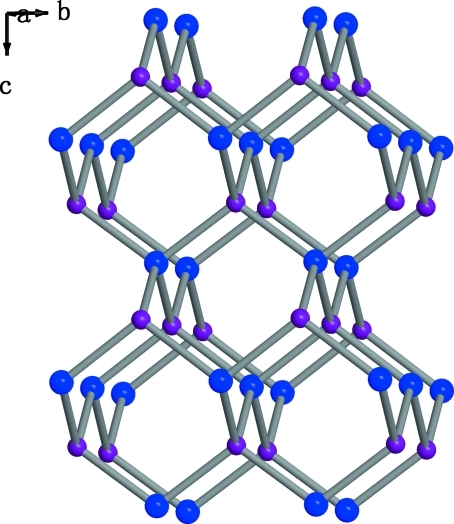
Topological figure for the network of (Ga0.71B0.29)PO4, oxygen atoms were omitted for clarity. Ga(B) atoms: blue spheres, P atoms: purple spheres.

## supplementary crystallographic information

### Comment

The high-cristobalite boron phosphate has long been used as an effective catalyst for various organic reactions such as hydration, dehydration, oligomerization (Moffat, 1978; Moffat & Schmidtmeyer, 1986; Morey *et al.*, 1983; Tada *et al.*, 1987; Tartarelli *et al.*, 1970). The catalytic activities depend on the ratio of P/B and surface area. In the case of excess B content, BPO_4_ catalysts consist predominately of Lewis acid sites, and show catalytic efficiencies for the dehydration. In contrast, in a region consisting of excess phosphorus P content, BPO_4_ catalysts have more Brønsted acid sites and exhibit catalytic activities for hydration. Applying trivalent cations to partially substitute boron may vary the ratio of P/B and modify the catalytic property. The possibility of modifying the catalytic properties by varieties of P/B ratio and searching for new phases in the borophosphate system intrigue us to investigate systems containing larger trivalent metal cations. In our previous investigations, a series of compounds with boron partially substituted by transition metals, such as Mn, Fe, Co, Ni, and Cu, has been characterized with low cristobalite type structure (Mi *et al.*, 1999). When we applied a smaller trivalent element Ga to modify the BPO_4_, the occupancy of Ga is more than 50%, while less than 50% for transition metal compounds (*M* = Mn, Fe, Co, Ni, and Cu). In consequence, the structure of (Ga_0.71_B_0.29_)PO_4_ are high-cristobalite structure instead of low-cristobalite type structure.

The crystal structure of (Ga_0.71_B_0.29_)PO_4_ is isostructural with the tetragonal high-cristobalite structure (Schulze, 1934; Schmidt *et al.*, 2004) with space group *P*4 which is built from alternating (Ga, B)O_4_ and PO_4_ tetrahedra interconnected by sharing the common O-vertices, resulting in a three dimensional network with two dimensional 6-membered ring tunnels running along the *a*- and *b*-axis, respectively. Every TO_4_ (T = Ga(B), P) tetrahedron connects to four neighboring TO_4_ tetrahedra. There are three types of positions for T-atoms. (Ga, B)1 and (Ga, B)2 sit at 1*c* and 1*b*, while P at 2*g*. The long (Schläfli) notation for (Ga, B) nodes is 6_2_.6_2_.6_2_.6_2_.6_2_.6_2_, while 6_2_.6_2_.6_2_.6_2_.6_2_.6_2_ for the P nodes, giving the net symbol (6_2_.6_2_.6_2_.6_2_.6_2_.6_2_)_3_ which can be represented by the short symbol (6^6^)_3_.

The (Ga, B)1–O and (Ga, B)2–O bond distances are 1.7079 (5) Å and 1.6979 (5) Å in the (Ga, B)O_4_ tetrahedra which are significantly larger than the B–O bond value of 1.463 Å in BPO_4_ (Schmidt *et al.*, 2004), but smaller than the bond values of 1.829 Å for Ga–O bond in GaPO_4_ (Achary *et al.*, 2003), indicating that boron and gallium occupy the same position.  After refining both the atomic occupation number and displacement parameters, it results in the ratio of Ga:B = 0.71:0.29. In turn, the Ga:P is 1.42:2, which is quite good agreement with that (Ga:P = 3:4) in the reactants for obtaining the pure phase. The introduction of gallium in the compound led to the deformation of all the tetrahedra and quite anisotropic expansion of the structure which results in lowering symmetry from space group *I*4 of BPO_4_ to *P*4 for the new compound.

### Experimental

The title compound has been synthesized *via* high temperature solid state reaction method and the structure refined from X-ray powder diffraction data. A mixture of H_3_BO_3_, NH_4_H_2_PO_4_, and Ga_2_O_3_ with molar ratio of B:Ga:*P* = 12:3:4 was well ground and reacted first at 973 K for 4 h, then cooled down to room temperature and reground again, pressed into pellets and reacted at 1373 K for 8 h, at last shut down the furnace and cooled down to room temperature. The extra B_2_O_3_ in the products were washed out by hot water.

### Refinement

The cell parameters were obtained by least-square fits of the powder diffractometer data using silicon (*a* = 5.4308 Å) as an internal standard. Although the powder pattern and cell parameters are quite different from BPO_4_, the starting atomic positional parameters can still be derived from the prototype BPO_4_ (Schmidt *et al.*, 2004). During the initial refinement, the unreasonable negative thermal parameters for the B position are indicative of partial substitutions by Ga. The boron position then were assumed to be occupied by two kinds of atoms and the occupacies were allowed to vary during the subsequent refinements. Because it is difficult to refine both the occupation numbers and atomic displacement parameters at the same time, a two-step process was applied to refine the occupancy numbers and atomic displacement parameters. At the begining, all the atomic displacement parameters were set to one value to refine the occupancy number, then fixed the occupany number to refine the displacement parameters. Both processes were performed alternately several times till reasonable values for both atomic occupancies and displacement parameters were obtained. Due to the individual refinement, the standard deviations given by the program are much too small to be a realistic estimate of the uncertainty.

### Crystal data

**Table:**

(Ga_0.71_B_0.29_)PO_4_	*Z* = 2
*M**_r_* = 147.61	*F*(000) = 140.4
Tetragonal, *P*4	*D*_x_ = 3.084 Mg m^−^^3^
Hall symbol: P -4	Cu *K*α_1_, Cu *K*α_2_ radiation, λ = 1.540500, 1.544300 Å
*a* = 4.7343 (1) Å	*T* = 293 K
*c* = 7.0896 (4) Å	white
*V* = 158.90 (1) Å^3^	flat sheet, 10 × 10 mm

### Data collection

**Table:**

Rigaku-D/max automatic powder diffractometer	Data collection mode: reflection
graphite	Scan method: step
Specimen mounting: packed powder pellet	2θ_min_ = 15.03°, 2θ_max_ = 100.02°, 2θ_step_ = 0.01°

### Refinement

**Table:**

Least-squares matrix: full	8500 data points
*R*_p_ = 0.076	Profile function: Thompson *et al.* (1987); Finger *et al.* (1994); Stephens et al. (1999)
*R*_wp_ = 0.129	35 parameters
*R*_exp_ = 0.072	2 restraints
*R*(*F*^2^) = 0.07586	(Δ/σ)_max_ = 0.001
χ^2^ = 3.204	Background function: The background function is a cosine Fourier series with a leading constant term. I_b_= B_1_+ΣB_j_cos[P*(j-1)] (j=2-9), here P = 2θ, B_j_ (j = 1-9) values are given below: 1: 935.903 2: -1634.98 3: 1422.47 4: -1094.93 5: 681.394 6: -358.046 7: 116.953 8: -17.2104 9: -22.9558

### Fractional atomic coordinates and isotropic or equivalent isotropic displacement parameters (Å^2^)

**Table:**

	*x*	*y*	*z*	*U*_iso_*/*U*_eq_	Occ. (<1)
Ga1	0.5	0.5	0.0	0.0347 (12)*	0.7002 (4)
Ga2	0.0	0.0	0.5	0.0406 (11)*	0.7179 (3)
P1	0.5	0.0	0.7456 (5)	0.0447 (12)*	
O1	0.7299 (3)	0.1326 (3)	0.6304 (5)	0.0493 (15)*	
O2	0.6286 (3)	0.7718 (4)	0.8669 (5)	0.0511 (16)*	
B1	0.5	0.5	0.0	0.0347 (12)*	0.2998 (4)
B2	0.0	0.0	0.5	0.0406 (11)*	0.2821 (3)

### Geometric parameters (Å, °)

**Table:**

(Ga/B)1—P1^i^	2.9759 (8)	(Ga/B)2—O1^ix^	1.6979 (5)
(Ga/B)1—P1^ii^	2.9759 (8)	(Ga/B)2—O1^x^	1.6979 (5)
(Ga/B)1—P1^iii^	2.9759 (8)	P1—O1	1.499 (3)
(Ga/B)1—P1^iv^	2.9759 (8)	P1—O1^ix^	1.499 (3)
(Ga/B)1—O2^i^	1.7079 (5)	P1—O2^xi^	1.509 (3)
(Ga/B)1—O2^iii^	1.7079 (5)	P1—O2^xii^	1.509 (3)
(Ga/B)1—O2^v^	1.7079 (5)	B1—O2^i^	1.7079 (5)
(Ga/B)1—O2^vi^	1.7079 (5)	B1—O2^iii^	1.7079 (5)
(Ga/B)2—P1^vii^	2.9386 (8)	B1—O2^v^	1.7079 (5)
(Ga/B)2—P1	2.9386 (8)	B1—O2^vi^	1.7079 (5)
(Ga/B)2—P1^viii^	2.9386 (8)	B2—O1^vii^	1.6979 (5)
(Ga/B)2—P1^iii^	2.9386 (8)	B2—O1^iii^	1.6979 (5)
(Ga/B)2—O1^vii^	1.6979 (5)	B2—O1^ix^	1.6979 (5)
(Ga/B)2—O1^iii^	1.6979 (5)	B2—O1^x^	1.6979 (5)
			
O2^i^—(Ga/B)1—O2^iii^	107.765 (2)	O2^xi^—P1—O2^xii^	110.502 (4)
O2^i^—(Ga/B)1—O2^v^	112.941 (3)	(Ga/B)2^xiii^—O1—P1	133.541 (1)
O2^i^—(Ga/B)1—O2^vi^	107.765 (2)	P1—O1—B2^xiii^	133.541 (1)
O2^iii^—(Ga/B)1—O2^v^	107.765 (2)	(Ga/B)1^xiv^—O2—P1^xv^	135.267 (1)
O2^iii^—(Ga/B)1—O2^vi^	112.941 (3)	P1^xv^—O2—B1^xiv^	135.267 (1)
O2^v^—(Ga/B)1—O2^vi^	107.765 (2)	O2^i^—B1—O2^iii^	107.765 (2)
O1^vii^—(Ga/B)2—O1^iii^	107.239 (2)	O2^i^—B1—O2^v^	112.941 (3)
O1^vii^—(Ga/B)2—O1^ix^	114.035 (3)	O2^i^—B1—O2^vi^	107.765 (2)
O1^vii^—(Ga/B)2—O1^x^	107.239 (2)	O2^iii^—B1—O2^v^	107.765 (2)
O1^iii^—(Ga/B)2—O1^ix^	107.239 (2)	O2^iii^—B1—O2^vi^	112.941 (3)
O1^iii^—(Ga/B)2—O1^x^	114.035 (3)	O2^v^—B1—O2^vi^	107.765 (2)
O1^ix^—(Ga/B)2—O1^x^	107.239 (2)	O1^vii^—B2—O1^iii^	107.239 (2)
O1—P1—O1^ix^	113.942 (3)	O1^vii^—B2—O1^ix^	114.035 (3)
O1—P1—O2^xi^	108.506 (2)	O1^vii^—B2—O1^x^	107.239 (2)
O1—P1—O2^xii^	107.696 (2)	O1^iii^—B2—O1^ix^	107.239 (2)
O1^ix^—P1—O2^xi^	107.696 (2)	O1^iii^—B2—O1^x^	114.035 (3)
O1^ix^—P1—O2^xii^	108.506 (2)	O1^ix^—B2—O1^x^	107.239 (2)

Symmetry codes: (i) *x*, *y*, *z*−1; (ii) *x*, *y*+1, *z*−1; (iii) *y*, −*x*+1, −*z*+1; (iv) *y*+1, −*x*+1, −*z*+1; (v) −*x*+1, −*y*+1, *z*−1; (vi) −*y*+1, *x*, −*z*+1; (vii) *x*−1, *y*, *z*;  (viii) *y*, −*x*, −*z*+1; (ix) −*x*+1, −*y*, *z*; (x) −*y*, *x*−1, −*z*+1; (xi) *x*, *y*−1, *z*; (xii) −*x*+1, −*y*+1, *z*; (xiii) *x*+1, *y*, *z*; (xiv) *x*, *y*, *z*+1; (xv) *x*, *y*+1, *z*.
